# Optimal Device Independent Quantum Key Distribution

**DOI:** 10.1038/srep30959

**Published:** 2016-08-03

**Authors:** S. Kamaruddin, J. S. Shaari

**Affiliations:** 1 Faculty of Science, International Islamic University Malaysia (IIUM), Jalan Sultan Ahmad Shah, Bandar Indera Mahkota, 25200 Kuantan, Pahang, Malaysia

## Abstract

We consider an optimal quantum key distribution setup based on minimal number of measurement bases with binary yields used by parties against an eavesdropper limited only by the no-signaling principle. We note that in general, the maximal key rate can be achieved by determining the optimal tradeoff between measurements that attain the maximal Bell violation and those that maximise the bit correlation between the parties. We show that higher correlation between shared raw keys at the expense of maximal Bell violation provide for better key rates for low channel disturbance.

Quantum cryptography, which is often a reference to the more specific study of quantum key distribution (QKD) had been developed as a secure way of distributing or establishing secure keys between parties^1^. While the security is based on information theoretic definitions (as opposed to modern cryptography with security based on computation complexity), the operational ingredient of such a cryptographic system would lie in the use of quantum systems as information carriers, thus setting definite constraints of quantum physics on information theoretic tasks.

Despite the various security proofs afforded thus far, the most pessimistic demands would not be satisfied as these proofs rely strongly on the requirement that the exploited degrees of freedom lies within the control of the legitimate users. Relaxing such a requirement has led to the birth of “device independent QKD” (DIQKD). The basis for security guarantee of this framework lies in the establishment of nonlocal correlations; a correlation that cannot be reproduced by any local theory. Such security feature in which can be observed from the violation of Bell-inequality^2^, assures that the output produced would still retain some amount of secrecy despite not having any prior knowledge of its internal process. The possibility of exploiting the nonlocal resource as a security measures was initially highlighted by ref. 3 though it would be ref. 4 that points out its potential in a device independent context. The preliminary work in the direction of DIQKD was first proposed by demonstrating proofs of security against an eavesdropper constrained by the no-signaling principle^5^. The no-signaling condition states that the marginal probabilities for any subset of the parties, say Alice and Bob, are independent of Eve’s measurement choice *z* (with measurement result *e*)^6^:


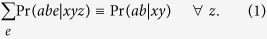


Though the protocol is proven to be inefficient, it follows from this idea that ref. 7 proposed the CHSH protocol (in which further detail was given in ref. 8) that is secure against an individual attack by an adversary who is supra-quantum, i.e. not limited by the dictates of quantum theory though bounded by the non-signaling principle. The individual attack strategy requires Eve, the adversary, to distribute a mixture of deterministic strategies and a nonlocal box, given by the Popescu-Rohrlich (PR) box^9^ to the legitimate parties.

One of the main setbacks regarding the CHSH protocol however, is its immediate implementation given Alice and Bob’s quantum framework. Such a protocol sees the legitimate users setting their choice of measurements to achieve a maximal CHSH violation for which any subset to be used for sharing a common string would, inherently carry errors due to non-overlapping basis. It is actually quite obvious to note that measurements to maximally estimate the nature of correlations for a bipartite entangled state; i.e. local or otherwise, is not compatible with measurements which would extract the maximally possible amount of correlation. This can be immediately seen as follows: If Alice and Bob wished to extract the maximal number of bits per-entangled pair by local measurements on each half of the pair, then every round of measurement requires them to have identical measurement bases and thus would not enable a determination of the type of correlation involved with certainty as evident from eq. (4) where subscribing to maximally overlapping bases results in the maximal value being 2. On the other hand, any measurement to ascertain the maximal possible local violation with certainty would not allow for Alice and Bob to share an error free string; the CHSH protocol is in fact an immediate example of this.

In ref. 10, a possible implementation which, while does not subscribe to a maximal CHSH violation, does nevertheless allow for a secret key to be established; as a matter of fact it was shown to exceed the CHSH protocol under a noiseless channel scenario. In this work, we shall consider in detail such protocols and determine their optimality. In effect, we will work with a binary measurement QKD for two parties, Alice and Bob, where each party would commit to either one of two measurement basis and each yields only binary results (contrary to ref. 6 in which Alice would be given the freedom to choose between 3 measurement bases instead of only two). The measurements settings would of course subscribe to quantum formalism. We will then consider two different scenario of how subsequent classical distribution of information between the legitimate parties, thus defining the protocol may allow for different secure key rates to achieve the highest possible.

## Results

### Binary Measurement QKD

We begin with a description of the protocol, which we define within a framework as described by quantum physics. Let Alice submit to Bob a quantum state of which each party would measure subsystems thereof available to them. In an ideal setup, we assume that this would result in Alice and Bob sharing the following maximally entangled states (a depolarizing channel would result in a Werner state^11^):


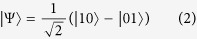


In each run, Alice and Bob can independently choose to apply one of two measurements with each choice resulting in binary outcomes. For definiteness, we describe Alice’s and Bob’s measurements as *x* and *y* with *x*, *y* ∈ {0, 1} and the binary results for their measurement choices are *a*, *b* ∈ {0, 1}, respectively.

Restricting measurements to projecting states on the *X* –*Z* plane of the Bloch sphere, any measurement can be described as projecting into the following states;


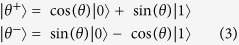


and we set *x* = 0 to be in the *Z* basis i.e. *θ* = 0 and *x* = 1 indicate the measurement made in angle *θ* = *α*. Meanwhile, Bob’s setting is described such that *y* = 0 and *y* = 1 correspond to measurement angles *θ* = *β* and *θ* = *γ*, respectively.

At the end of the transmission and measuring process, Alice and Bob would exchange classical information to allow them to share a raw key. The simplest scenario is that of ref. 8 in which only Alice would reveal her measurement bases over a public channel and Bob would commit to flipping bits in selected cases to maximize correlations with Alice for key purposes (we refer to this as Version I). Another scenario (which we refer to as Version II) would be for both to disclose their bases (as in ref. 10) and a raw key is defined by the bits derived from the measurement set *x* = *y* = 0. In both cases, the parties will determine the security of the protocol by means of checking for violation of Bell inequality^2^ on a subset of the measurement results. In this work we will consider the case where Alice and Bob would compute the amount of the following CHSH correlations^12^:


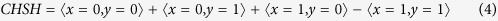


in which local correlations is bounded by inequality −2 ≤ *CHSH* ≤ 2.

However, in modeling a noisy setting, we shall assume a depolarizing channel between the legitimate parties and thus, (2) is transformed to


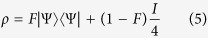


where 0 ≤ *F* ≤ 1 with *F* = 1 represent the noise-free condition. From the results obtained when measuring state *ρ* (see Table 1), it is not difficult to show that the estimation of CHSH violation (4) can be written as


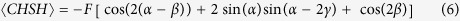


Depending on the results, Alice and Bob may choose to abort the protocol or proceed to error correction and privacy amplification.

### Security Analysis: Supra-quantum Eve

We consider the pessimistic view where Eve has control of the degrees of freedom of Alice and Bob’s observables. We could imagine that the eavesdropper, Eve fabricated the devices and she is in fact controlling the source. The legitimate parties are essentially ignorant of the internal process of the protocol and their devices may be regarded as black boxes with binary inputs and outputs. We define Eve’s strategy as being constrained by the no-signaling principle while requiring observations made by both Alice and Bob to be consistent with quantum predictions.

Similar to the CHSH protocol^7^,^8^, Eve’s strategy is to submit to Alice and Bob a convex combination of probabilistic distributions of deterministic and nonlocal strategies. A deterministic strategy is a strategy for which results obtained for any given set of Alice’s and Bob’s measurement would be fully determined (i.e no uncertainty and conforms completely to a local theory)^13^. On the other hand, a nonlocal strategy is one in which a PR box is distributed and measurement results are not only probabilistic, but also violates the CHSH inequality up to its algebraic maximum^13^. However, since our protocol is described in terms of an anti-correlated state (2) (like in ref. 10), it would be appropriate to use the anti-PR (aPR)^14^ box for which all measurement settings (except for *x* = *y* = 1) result in anti-correlations rather than the PR box that provides for correlations. The aPR box, which is equivalent to the PR box up to a trivial local processing^13^, violates the lower bound of CHSH (as opposed to the PR box violating on the positive side) is given by the probability function,


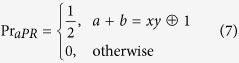


where ⊕ is addition modulo 2. The deterministic strategies are described by four deterministic functions *G*:[4] × {0, 1} → {0, 1} for *r* = 1, 2, 3, 4 defined by


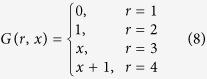


Thus, the sixteen deterministic strategies are given by 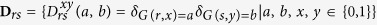 where 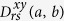 gives the probability of having input *x*, *y* resulting in output *a*, *b* for strategy *rs*^13^. However, like in ref. 10, we are only interested in the following eight deterministic strategies, **D**_12_, **D**_14_, **D**_21_, **D**_23_, **D**_32_, **D**_33_, **D**_41_, **D**_44_ which would saturate the local bound on the negative side of the CHSH range. Eve’s strategy and her information on Alice-Bob distribution can be summarized in Table 2 which is a ‘complimentary’ table to that in ref. 8 where Eve would use a PR box^9^ instead. Note that the symbol *p*_*rs*_ represent the probability of sending strategy **D**_*rs*_ and *p*_*NL*_ is the probability of sending aPR box. While it remains unclear if this strategy should be the most general one available to Eve, we feel that there may be some reasonability for this choice; this is in view of the argument made in ref. 10 for Eve’s distribution of a two-party non signaling correlation instead of a possible three-party scenario where the latter results in two out of three parties being totally uncorrelated. It would certainly be interesting to consider a complete proof for the optimality of such attack schemes for a supra-quantum Eve though this would be outside the scope of the current manuscript.

With aPR box violating the CHSH inequality up to its algebraic minimum value of −4, the estimation of local correlation, 〈*CHSH*〉 that Alice and Bob may find would be


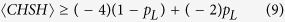


in which *p*_*L*_ = 1 − *p*_*NL*_ with *p*_*L*_ = *p*_12_ + *p*_14_ + *p*_21_ + *p*_23_ + *p*_32_ + *p*_33_ + *p*_41_ + *p*_44_. In the ensuing sections, the security analysis, given Eve’s attack is constructed within the framework of an eavesdropper who may be supra-quantum but would emulate Alice and Bob’s expectations; i.e. the statistics of their measurement results must be consistent with the expectation of quantum physics. We thus assume a one-to-one correspondence rule from the set of Eve’s probabilities of strategies sent, *E*_*ijkl*_, where *i*, *k* and *j*, *l* are Alice and Bob’s measurement settings and results respectively to the set of probabilities of Alice-Bob’s measurements, Pr(*a* = *i*, *b* = *j*|*x* = *k*, *y* = *l*).

### Version I

We consider the simplest case where only one party, say Alice, would publicly disclose her measurement bases. To ensure that Eve would be at a disadvantage in regards to the correlations between Alice and Bob, i.e. to ensure the correlations are derived from strategies that should include the nonlocal box, referring to Table 2, we consider the stipulation where Bob would flip all his bits except in the event where Alice declares *x* = 1 and Bob measure *y* = 1. This step is equivalent to the pseudosifting procedure introduced by ref. 8 (the main concern there was to maximize the correlations between Alice and Bob). The error rate for Alice and Bob, 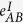 originates from Eve’s sending strategies, which after pseudoshifting is given by the probability, 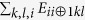. In terms of of the angles *α*, *β*, and *γ*, we refer to the one-to-one correspondence between the legitimate parties’ measurement settings and the probabilities of Eve’s strategies (Tables 1 and 2 respectively) and the error rate is then given by


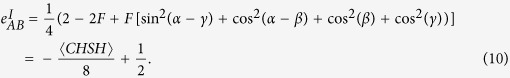


For each deterministic strategy, Eve would only learns about one of Alice’s setting, while being totally ignorant about the other. This is described in detail in ref. 8, and assuming the choice of measurement basis is equiprobable, Eve’s information gain on Bob would then be


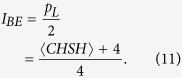


From eq. (10) and eq. (11), the key rate, *K*_*I*_ is then given by^15^


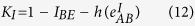


with the binary entropic function *h*(*p*) = −*plog*_2_*p* − (1 − *p*)*log*_2_(1 − *p*). It is obvious that the secret key rate is a monotonically increasing function of the CHSH violation (it is clear from eq. (10) that an increase in 〈*CHSH*〉 would decrease the uncertainty between Alice and Bob) and thus maximized for angles *α*, *β*, *γ* maximizing the CHSH violation and the protocol would be the CHSH protocol^7^,^8^. This could be actually derived from eq. (25) in ref. 8 where in a quantum setup, Alice and Bob prescribe measurements that would maximize the Bell violation. Thus we can conclude that generalizing the angles of measurements, in a case where only Alice reveals her measurement bases, the most optimal protocol would necessarily reduce to that of CHSH protocol^7^,^8^.

### Version II

In this version, we require that both Alice and Bob reveal their measurement bases, and bits for key purposes would be extracted from the case *x* = *y* = 0. The error in the strings that Alice and Bob would have to correct, 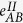 (corresponding to Eve’s strategy 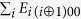) is given by,


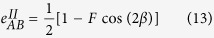


As Alice’s and Bob’s measurements’ settings are eventually made known, any measurement coinciding with the receipt of Eve’s deterministic strategies would provide the latter with complete information. Given that, Eve’s information gain, *I*_*AE*_ = *p*_*L*_ and along with eq. (9) the key rate, *K*_*II*_ can be shown to be,


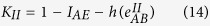



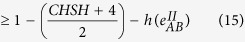


in which


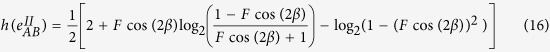


It should be noted that, as Alice’s and Bob’s measurement bases are randomly chosen, the actual fraction of bits that go into *K*_*II*_ from the total number of runs would be less than 1 (in fact if the choices were equiprobable, then the case *x* = *y* = 0 would occur only 1/4 of the time). However, given that the cases when *x*, *y* = 1 are not used for raw key purposes, i.e only for checking a CHSH violation (along with a sample for when *x*, *y* = 0), similar to ref. 6, one can imagine having a bias in bases’ choice, and so long as sufficient statistics is achieved towards determining CHSH violation, one can have the probability for *x* = *y* = 0 approaching 1. In maximizing the key rate, we consider the following partial derivatives;


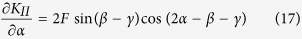



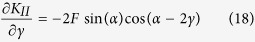



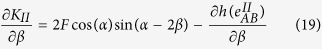


where


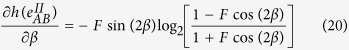


Considering eq. (4), it is obvious that measurement choices such {*x* = 0} = {*x* = 1} or {*y* = 0} = {*y* = 1} would result in no violation of the CHSH inequality no matter the given bipartite state. Thus, *α* ≠ 0 and *β* ≠ *γ* and equating the partial derivatives of *K*_*II*_ to zero, we find *α* − 2*γ* = *π*/2 + *I*_1_ and 2*α* − *β* − *γ* = *π*/2 + *I*_2_ where *I*_1_ and *I*_2_ are non-negative integers. Solving this gives us


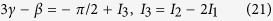


**Figure 1:**
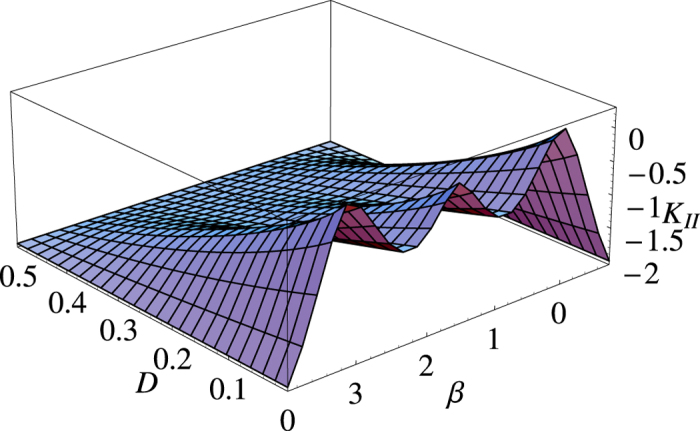
Key rate, *K*_*II*_ for varying *β* and *D*.

Thus a choice of one variable, say *β* determines all other angles. By defining *F* = 1 − 2*D*, such that the disturbance, *D* represent the probability that the measurement results from the same basis agree, we can see from Fig. 1, a plot of the secure key rate for varying *β* (for simplicity we choose *I*_1_ = *I*_2_ = 0).

**Figure 2:**
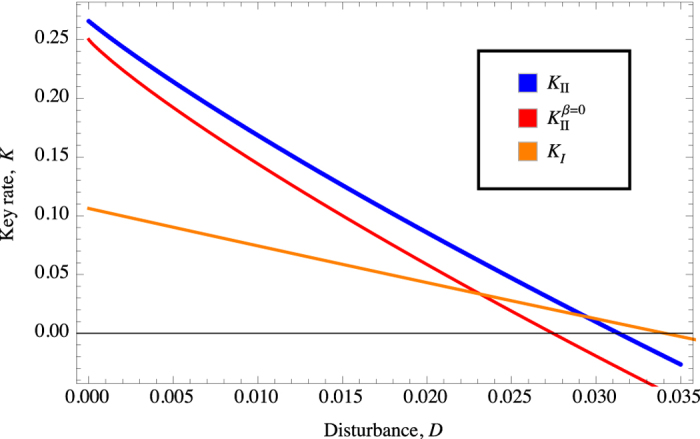
Key rate as a function of disturbance, *D*. (**a**) *K*_*II*_ represent numerically optimised key rate (**b**) 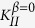 denote the extracted key rate given that *β* = 0 (**c**) *K*_*I*_ indicate the key rate achievable by CHSH protocol^7^,^8^ without postprocessing.

An analytical solution is unfortunately not immediate; and we plot a numerically optimised secure key rate in Fig. 2. While it is the case that a different value for disturbance, *D*, would require a different set of angles used, this may be not too practical as one must commit to determining *D* prior to choosing the angles. It is possibly simpler to decide on one fixed value of *β* (thus the other angles as well) and derive a secure key for every possible *D*. We could simplify matters greatly by considering the choice in ref. 10 of *β* = 0, and letting *I*_1_ = *I*_2_ = 0. We then have *γ* = −*π*/6 and *α* = *π*/6. In order to show that a maximal key rate is in fact achievable with such angles for *β* = 0, we consider the Hessian matrix, *H* which is given by


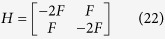


From eq. (22), we can easily see that 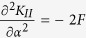 and |*H*| = 3*F*^2^. Since *F* does not take on a negative value and *F*^2^ will always be positive then we can deduce that 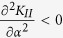 and |*H*| > 0 thus implying that a maximal key rate is achievable when *γ* = −*π*/6 and *α* = *π*/6 given *β* = 0.

## Discussion

We compare the performance of the protocols of Version I and II in Fig. 2. We can immediately observe that the protocol of Version II (for varying *β* and *β* = 0) outperforms Version I for *D* up to about 3% and 2.4% respectively when the terms related to error correction (in terms of Alice-Bob mutual information) play a more prominent role in determining the maximal achievable key rate as opposed to privacy amplification. In general, this can be understood in the context of the legitimate parties making measurements to maximise correlations between them at the expense of determining the actual amount of local violation their bits are derived from. On the other hand, for larger values of *D*, the information that Eve gleans from Bob becomes more pronounced for Version II; where Alice and Bob have little information on the type of correlation they actually share. We can in fact, in this vein, write an inequality to denote when the secure key rate of one protocol, *K*_*II*_ would exceed another, *K*_*I*_ in terms of the difference of mutual information between the protocols.


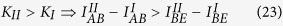


where 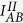 and 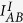 are the mutual information between Alice-Bob for protocols I and II respectively while, 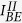 and 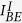 are Eve’s information gain for protocols I and II respectively.

**Figure 3:**
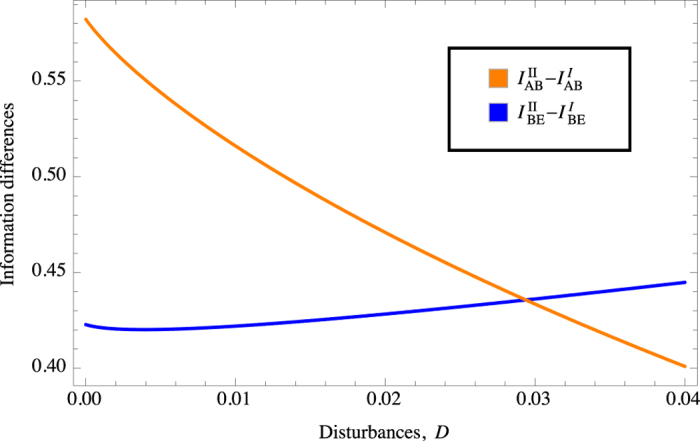
Differences of Alice-Bob mutual information (orange curve) and Eve information gain (blue curve) between the protocols of Version I and Version II versus *D*.

We see from Fig. 3 in fact such an inequality holds only up to *D* ≈ 0.03 where errors between the two legitimate parties become less important in determining the key rate as the difference between the two versions decrease while the difference in Eve’s gain increases.

The case for the protocol Version II with *β* = 0 against Version I is similar and applying inequality of eq. (23) gives


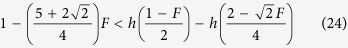


so long as *D* < 2.4% (this can be checked through simple numerics for eq. (24)). The fact that the protocol of Version II for varying *β* exceeds that of *β* = 0 is rather obvious from the fact that the former is based on the optimal choice for *β*.

## Conclusion

In the search for an ultimately secure key distribution procedure with the most pessimistic assumptions, protocols based on violating Bell inequalities were conceived. Limiting an adversary, Eve, with only the no-signaling principle while being supra-quantum still nevertheless allows for secure key distribution to be established. However, in this work we have noted that deriving a secure key and determining a Bell violation are clearly two incompatible processes; one can only be achieved maximally at the expense of the other and thus generating the most optimal secure key rate must necessarily capitalise on a possible trade-off.

In this work, we have considered two variants of a QKD protocol where the basic building block would really be two parties committing to measurements, each chosen from a set of two bases and each yielding binary results. Version I, which allows for the legitimate parties to make measurements with non overlapping bases and minimal disclosure of bases (by Alice only) provides for maximal determination of a Bell violation. This naturally results in the CHSH protocol^8^. It however, evidently sacrifices the actual correlation between the resulting shared raw key. Version II on the other hand allows for higher correlation between the shared raw key though at the expense of ascertaining a Bell violation; hence decreasing the legitimate parties’ ability to determine how secure their key is from Eve and effectively resulting in more bits to be discarded in privacy amplification. We have also used a simpler form of Version II by having a maximal correlation between Alice and Bob in one set of bases’ choice (setting *β* = 0). On the whole, we note that Version II exceeds Version I for disturbance on the channel for up to about 3% and 2.4%, the latter is for the case *β* = 0. The latter may provide for ease for practical implementation due to having a fixed set of measurement bases for any disturbance on the channel while Version II on the whole is better suited for the low channel disturbance.

## Additional Information

**How to cite this article**: Kamaruddin, S. and Shaari, J. S. Optimal Device Independent Quantum Key Distribution. *Sci. Rep.* **6**, 30959; doi: 10.1038/srep30959 (2016).

## Figures and Tables

**Table 1:** The correlations table as a result of measuring state *ρ*.

	*y* = 0, *b* = 0	*y* = 0, *b* = 1	*y* = 1, *b* = 0	*y* = 1, *b* = 1
*x* = 0, *a* = 0	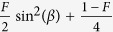	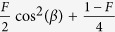	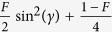	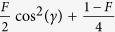
*x* = 0, *a* = 1	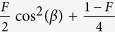	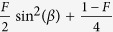	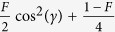	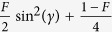
*x* = 1, *a* = 0	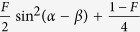	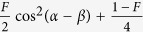	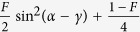	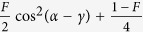
*x* = 1, *a* = 1	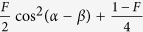	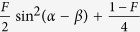	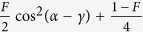	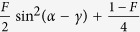

**Table 2:** Table showing probability distribution of Eve sending the corresponding strategy (as shown in the parentheses) to Alice and Bob.

	*y* = 0, *b* = 0	*y* = 0, *b* = 1	*y* = 1, *b* = 0	*y* = 1, *b* = 1
*x* = 0, *a* = 0	*p*_33_ (**D**_33_)	*p*_*NL*_/2 (*P*_*aPR*_)*p*_12_ (**D**_12_)*p*_14_ (**D**_14_)*p*_32_ (**D**_32_)	*p*_14_ (**D**_14_)	*p*_*NL*_/2 (*P*_*aPR*_)*p*_12_ (**D**_12_)*p*_32_ (**D**_32_)*p*_33_ (**D**_33_)
*x* = 0, *a* = 1	*p*_*NL*_/2 (*P*_*aPR*_)*p*_21_ (**D**_21_)*p*_23_ (**D**_23_)*p*_41_ (**D**_41_)	*p*_44_ (**D**_44_)	*p*_*NL*_/2 (*P*_*aPR*_)*p*_21_ (**D**_21_)*p*_41_ (**D**_41_)*p*_44_ (**D**_44_)	*p*_23_ (**D**_23_)
*x* = 1, *a* = 0	*p*_41_ (**D**_41_)	*p*_*NL*_/2 (*P*_*aPR*_)*p*_12_ (**D**_12_)*p*_14_ (**D**_14_)*p*_44_ (**D**_44_)	*p*_*NL*_/2 (*P*_*aPR*_)*p*_14_ (**D**_14_)*p*_41_ (**D**_41_)*p*_44_ (**D**_44_)	*p*_12_ (**D**_12_)
*x* = 1, *a* = 1	*p*_*NL*_/2 (*P*_*aPR*_)*p*_21_ (**D**_21_)*p*_23_ (**D**_23_)*p*_33_ (**D**_33_)	*p*_32_ (**D**_32_)	*p*_21_ (**D**_21_)	*p*_*NL*_/2 (*P*_*aPR*_)*p*_23_ (**D**_23_)*p*_32_ (**D**_32_)*p*_33_ (**D**_33_)

This is a ‘complimentary’ table to that in ref. 8 where Eve would use a PR box instead.
